# Solar Flare 1/f Fluctuations from Amplitude-Modulated Five-Minute Oscillation

**DOI:** 10.3390/e25121593

**Published:** 2023-11-28

**Authors:** Masahiro Morikawa, Akika Nakamichi

**Affiliations:** 1 Department of Physics, Ochanomizu University, 2-1-1 Otsuka, Bunkyo, Tokyo 112-8610, Japan; 2 General Education, Kyoto Sangyo University, Motoyama Kamigamo Kita-ku, Kyoto 603-8555, Japan; nakamichi@cc.kyoto-su.ac.jp

**Keywords:** 1/f fluctuations, solar flare, solar five-minute oscillation, resonance, amplitude modulation

## Abstract

We first report that the solar flare time sequence exhibits a fluctuation characterized by its power spectral density being inversely proportional to the signal frequency. This is the 1/f fluctuation, or pink noise, observed ubiquitously in nature. Using GOES16 data, we found that low-energy flares ($E\leq E_{mean}$) display 1/f fluctuations, whereas high-energy flares ($E>E_{mean}$) show a flat spectrum. Furthermore, we found that the timing sequence of the flares reveals clearer 1/f fluctuations. These observations suggest that the solar flare 1/f fluctuations are associated with low-energy phenomena. We investigated the origin of these 1/f fluctuations based on our recent hypothesis: 1/f fluctuations arise from amplitude modulation and demodulation. We propose that this amplitude modulation is encoded by the resonance with the solar five-minute oscillation (SFO) and demodulated by magnetic reconnections. We partially demonstrate this scenario by analyzing the SFO eigenmodes resolving the frequency degeneration in the azimuthal order number *m* using the solar rotation and resonance. Given the robust nature of 1/f fluctuations, we speculated that the solar flare 1/f fluctuations may be inherited by the various phenomena around the Sun, such as the sunspot numbers and cosmic rays. In addition, we draw parallels between solar flares and earthquakes, both exhibiting 1/f fluctuations. Interestingly, the analysis applied to solar flares can also be adapted to earthquakes if we read the SFO as Earth’s free oscillation and magnetic reconnections as fault ruptures. Moreover, we point out the possibility that the same analysis also applies to the activity of a black hole/disk system if we read the SFO as the quasi-periodic oscillation of a black hole.

## 1. Introduction

A solar flare is a sudden energy eruption in the solar atmosphere [1]. It is triggered by magnetic reconnections, and the enormous magnetic energy of $10^{17}$–$10^{26}$ J is converted into plasma particle acceleration, heating, and light emission. Solar flares are complex phenomena, and the statistical approach to predicting them is effective, as in the case of earthquakes, which are sudden energy eruptions of the Earth’s crust.

It is well known that solar flares and earthquakes are similar to each other, and they show similar statistical properties. In particular, the scaling relation laws, such as the Gutenberg–Richter law [2] and the Omori law [3], are universal laws for both solar flares and earthquakes [4,5].

Here, in this study, we concentrated on solar flares and aimed to add one more universal scaling law in the ultra-low-frequency region of the power spectral density (PSD) for the long time sequence of solar flares. It was revealed that the solar flare time sequence shows a power law that is almost inversely proportional to the frequency in the PSD. This is often called the 1/f fluctuation or pink noise and appears in most fields of nature and human activities [6,7]. However, the origin of this fluctuation has not been clarified despite a large number of studies performed in the past century.

We recently proposed that the general origin of the 1/f fluctuation is amplitude modulation (AM), or the beat of many waves with accumulating frequencies [8]. In particular, this frequency accumulation is possible in resonance, where many eigenfrequencies are systematically concentrated in a narrow domain.

Applying this method in [9], we studied the 1/f fluctuations in seismic activity. The seismic-energy time sequence shows an apparent 1/f fluctuation in its PSD by more than three digits if giant earthquakes are excluded. Therefore, seismic 1/f fluctuations are considered to be associated with low-energy phenomena. In this case, perpetually exciting Earth’s free oscillation (EFO) in the lithosphere results in resonance, which yields an AM or wave beats. A relatively low-energy EFO will sufficiently trigger the fault eruption and cause earthquakes.

In this study, applying this proposal to solar flares, we aimed to verify our proposal and determine the statistical properties of complex systems in general. When we naively used all the energy time series data, we obtained almost a flat PSD at low-frequency regions and discerned no clear pink noise. However, if we restricted the solar flare events to having their energy below the mean, we obtained clear pink noise. Therefore, we speculated that the 1/f fluctuation in solar flares is associated with low-energy phenomena. Interestingly, this situation is the same as for seismic activity, as explained above.

The resonance in the solar case would be characterized by solar five-minute oscillations (SFOs), which are perpetually excited by the turbulence in the solar convective region [10,11]. The eigenfrequencies have been precisely measured and calculated by many studies assuming appropriate solar models. Using these observational data, we constructed the superposition of many waves with these eigenoscillations, including the fine splitting structure using the solar rotation and the resonance effects. Then, we could obtain 1/f fluctuations from the thresholding of these data in the PSD. Thus, we can partially demonstrate the AM theory for 1/f fluctuations in solar flares.

Since pink noises often appear in various fields of science, we explored the neighboring phenomena to the solar flares. Then, we found 1/f fluctuations in several phenomena such as solar winds, sun spot numbers, and some terrestrial traces. These facts may indicate the robustness of pink noises.

This paper is organized as follows. In Section 2, we explore the GOES data and RHESSI data of solar flares and analyze the PSD. In Section 3, we explain the AM proposal for the origin of 1/f fluctuations from resonance. In Section 4, we superpose the eigenmodes of the SFO and obtain pink noise. In Section 5, we study another statistical characterization of solar flares using the Weibull distribution and compare it with 1/f characterization. In Section 6, we emphasize the robustness of 1/f fluctuations being the origin of a variety of pink noises. We point out that the 1/f fluctuation property is inherited by solar winds, sunspot events, and some traces on Earth. In Section 7, we conclude our work and briefly describe the possible future research, including the back hole.

## 2. Solar Flare Fluctuations

Solar flares are eruptive energy-release events in the solar atmosphere. Each event transforms enormous magnetic energy into plasma particle acceleration, visible light, X-ray emission, and more. Our focus was specifically directed towards the soft X-ray flux data acquired by the GOES16 satellite from February 2017 to September 2023, covering a span of 6.6 years [12]. We mainly used xrsa1_flux and xrsa2_flux xrsa_flux.

**Figure 1:**
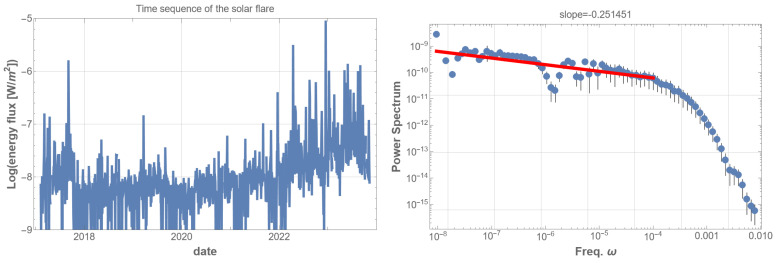
(**left**) Goes16 soft X-ray flux data from February 2017 to September 2023 (6.6 years) [12]. The vertical unit is $W/m^{2}$ in the logarithmic scale. Although the data show an apparent trend associated with the eleven-year solar activity cycle, we did not apply any artificial operation for our analysis, as extraction of the trend does not greatly affect the result. (**right**) Power spectral density (PSD) of the energy-flux time sequence of all the GOES16 solar flare data over 6.6 years. The time is measured in seconds, and the frequency unit is Hz. Since the time interval of the original data is not uniform, we redistributed the energy-flux data after making the time interval equal and assigned zero values for vacant intervals. Then, we applied the fast Fourier transformation to obtain the PSD. Various window functions were tested, but as they did not significantly alter the results, the data without any artificial arrangement were used. The Fourier-transformed data were averaged within the equal bins on a logarithmic scale. A red line in the low-frequency domain in the PSD graph represents the best fits of the data according to the least squares method within that domain. This procedure is the same for all PSD analyses below. This PSD illustrates that the energy time sequence is random, as indicated by the almost flat red line fitting the data points in the low-frequency domain.

Initially, we used all the time sequence data of the soft X-ray energy flux in units of $W/m^{2}$, as shown in Figure 1 (left). The corresponding PSD appears almost flat and random in the low-frequency regions, as depicted in Figure 1 (right). However, this is consistent with the previous research [13], in which the authors partially extracted the 1/f fluctuations in the GOES6 data by superposing multiple PSDs. We adopted another approach to extract the entirety of the 1/f fluctuations by restricting the energy flux.

**Figure 2:**
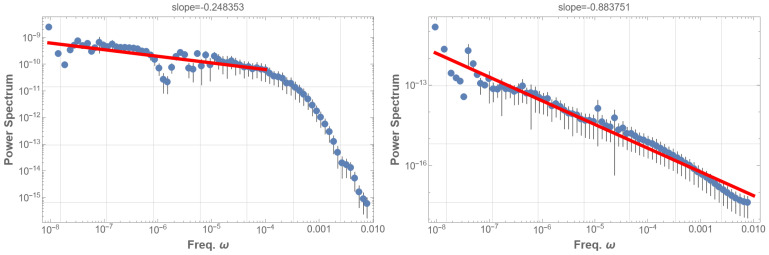
(**left**) PSD for the high-energy group, encompassing events with energy exceeding the mean. The PSD does not exhibit 1/f fluctuations, akin to Figure 1 (right). (**right**) PSD for the low-energy group, including events with energy below the mean. The PSD clearly shows 1/f fluctuation with an index of −0.88 over more than five orders of magnitude. Generally, 1/f fluctuation is defined by a power index of −1±0.5 in the PSD. A red line in the low-frequency domain in the PSD graph represents the best fits of the data according to the least squares method within that domain.

Subsequently, we analyzed two datasets: the high-energy group, which include all events with energy exceeding the mean ($4.4\times 10^{-8}$ $W/m^{2}$) (i.e., comprising part of class B, and classes C, M, and X), and the low-energy group, comprising events with energy below the mean (class A and part of class B). Figure 2 presents the PSDs for these two groups. The 1/f fluctuation is evident in Figure 2 (right), representing the low-energy data. Conversely, the high-energy group’s PSD as in Figure 2 (left), does not exhibit 1/f fluctuations and resembles the pattern in Figure 1 (right); high-energy data disrupt the 1/f fluctuations in the solar flare. These observations suggest that solar flare 1/f fluctuations are associated with low-energy phenomena.

**Figure 3:**
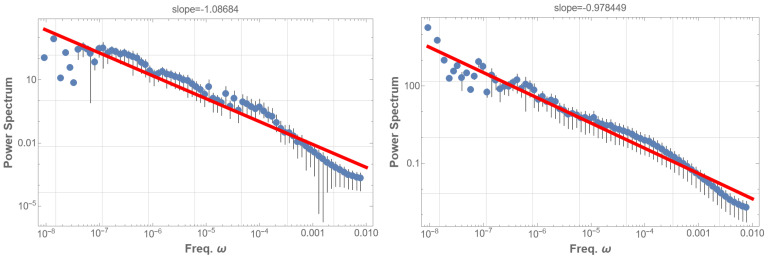
(**left**) Similar to Figure 1 (**right**), but with energy information entirely removed: following time-uniformization, finite energy values were reset to one, leaving the others at zero. This represents the time sequence of the flare occurrence, or the unweighted distribution. The PSD displays typical pink noise behavior with a power index of −1.1 in the frequency range $10^{-3}$ to $2\times 10^{-8}$ Hz. (**right**) Corresponding PSD for the high-energy group. This clear 1/f fluctuation, with a power index −0.98, contrasts with Figure 2, where all energy information is included, resulting in a flat spectrum. A red line in the low-frequency domain in the PSD graph represents the best fits of the data according to the least squares method within that domain.

To confirm that the solar flare 1/f fluctuation is independent of energy, we removed the energy information from the data: all energy values in the time sequence were set to one. The PSD for the entire dataset then displayed a 1/f fluctuation with a power index of −1.1 as shown in Figure 3 (left). Similarly, the high-energy group’s PSD, with energy information removed, exhibits a 1/f fluctuation with a power index of −0.98 as in Figure 3 (right).

All together, 1/f fluctuation with a power index of approximately −0.9∼−1.1 is observed within about five orders of frequencies, corresponding to timescales from about an hour ($10^{-3}$ Hz) to 6.6 years ($2\times 10^{-8}$ Hz). This 1/f fluctuation appears when high-energy solar flare events are excluded or when energy information is completely removed.

These findings suggest that the solar flare 1/f fluctuation does not reflect the energy scaling structure typically caused by the self-organized criticality (SOC) formed by energy cascades from small to large, although SOC may be crucial for explaining popular scaling laws like the Gutenberg–Richter and Omori laws. On the contrary, solar flare 1/f fluctuation seems to be a low-energy phenomenon, probably triggered by a tiny energy source. This point will be further discussed in the next section.

We also analyzed short-term GOES16 solar flare data for one week, chosen arbitrarily. The results are consistent, showing a 1/f fluctuation with an index of −1.2 across a frequency range of $2\times 10^{-3}$ to $2\times 10^{-6}$ Hz. This extends across the entire week, encompassing frequencies lower than the typical frequency of SFO.

So far, we have examined soft X-ray data from GOES observations, which provide one perspective of solar flares. We now turn to hard X-ray data from RHESSI observations for another perspective on solar flare.

**Figure 4:**
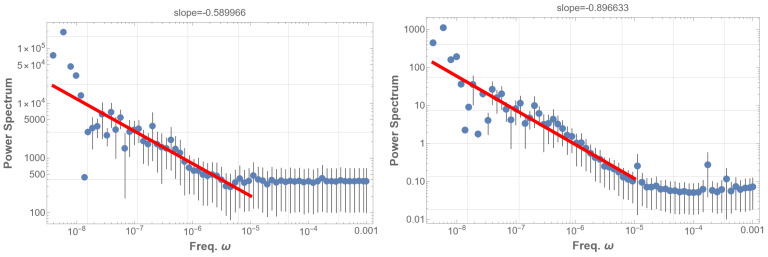
PSD analysis was conducted on the energy time sequence of RHESSI solar flare data spanning 16 years, from 2002 to 2018. The mean energy range spans from 4.5 keV to 3.9 MeV. A red line in the low-frequency domain in the PSD graph represents the best fits of the data according to the least squares method within that domain. (**left**) The PSD of the energy time sequence reveals a power behavior with an index of −0.59, where 1/f fluctuation is not distinctly apparent. (**right**) The PSD of the flare occurrence time sequence is presented. It displays a power behavior with an index of −0.90, clearly indicating the presence of 1/f fluctuation. Additionally, we analyzed the PSD of other indicators of the solar flare time sequence. Most of these indicators exhibited a power index around −0.40, with the notable exception of the duration, which demonstrates a power index of −0.88.

Analysis of 16 years of RHESSI solar flare data from 2002 to 2018 [14] mostly aligns with the GOES findings. The RHESSI solar flare energy time sequence displays a marginal 1/f fluctuation with an index of −0.59 as shown in Figure 4 (left). In contrast, the occurrence time sequence exhibits a pronounced 1/f fluctuation with an index of −0.90 within the frequency range of $10^{-5}$ to $2\times 10^{-9}$ Hz, as depicted in Figure 4 (right). This fluctuation spans from a day to the entire observation period, though data scattering at the lowest frequency domain is notable. Other flare indicators, such as total count, peak count rate, and duration times mean energy, were also examined. These indicators generally yielded a power index around −0.40, diverging significantly from 1/f fluctuations. However, the duration alone demonstrated a 1/f fluctuation with a power index of −0.88, with slightly less scattering than Figure 4 (right).

Finally, in our analysis of solar flares in this section, we acknowledge certain limitations. Each solar flare event has been characterized as instantaneous in our dataset. However, realistically, a solar flare involves substantial energy transfer from the magnetic field to plasma and particles over a finite duration; large flares may have prolonged durations, while smaller flares are more instantaneous. We have excluded the extended profiles, such as the decay phases of large flares. This decision is based on the fact that, in the 16-year RHESSI dataset, the average time interval between flares is 4865 s, while the average duration of a flare is 642 s, with a maximum of 4500 s. Consequently, we believe this approach does not significantly alter our conclusion that solar flare 1/f fluctuation is predominantly a low-energy phenomenon. This does not discount the high-energy core component of a solar flare. The relationship between the low-energy and high-energy components of solar flares, particularly in the context of SOC, will be further discussed in the next section.

What mechanism then gives rise to this universal 1/f fluctuation at the low-energy regime of various solar flare datasets?

## 3. Amplitude Modulation from Resonance

We recently proposed a potential origin for 1/f fluctuation, attributing it to wave beat or amplitude modulation [8]. Given the generality of this mechanism, our intention is to extend its application to the context of solar flare 1/f fluctuation within the scope of this paper.

The foundation of this theory rests on the observation that waves with accumulating frequencies yield robust low-frequency signals. In cases where this accumulation is systematic, such as in instances of resonance, synchronization, or infrared divergence, the wave beats consistently manifest a power law with an index approximately equal to −1. The ubiquity of this phenomenon in nature stems from the prevalence of simple physics: wave beat or amplitude modulation.

Consider an example of a beat: waves with frequencies of 440 Hz and 441 Hz yield a beat. In musical sounds, this beat is ‘audible’ as a sinusoidal amplitude oscillation with a frequency of 1 Hz. However, a Fourier transform of the original signal does not yield this 1 Hz signal, only the original two frequencies. To extract this encoded 1 Hz signal, one simple method is to square the data and then apply a Fourier transform. This approach allows for the extraction of the encoded low-frequency signal at 2 Hz, though it is twice the original frequency. Decoding is not limited to squaring but can also involve absolute value, rectification, fourth-order power, thresholding, or other methods, resulting in a variety of pink noise patterns.

Another example is AM radio, where amplitude modulation is employed. Using high-frequency radio waves between 526.5 kHz and 1606.5 kHz, a low-frequency audible signal is encoded. However, the encoded sound cannot be directly heard, as the rapidly oscillating positive and negative parts in the wave cancel each other out, leaving no audible signal. Demodulation is achieved by rectifying the radio wave signal, traditionally through germanium diodes or vacuum tubes. This process is indispensable for extracting the encoded low-frequency signal, such as 1/f fluctuation [8].

In the context of solar flares, we hypothesize that the resonant mode crucial for the manifestation of 1/f fluctuations is the solar five-minute oscillation (SFO), a phenomenon consistently activated within the solar atmosphere through turbulent convection [10,11]. Specifically, pressure modes of SFO exhibit accumulating eigenfrequencies, particularly converging towards lower angular indices *l*. We aim to examine whether this frequency accumulation effectively produces a 1/f power spectral density.

If SFO induces amplitude modulation, demodulation becomes imperative for observing 1/f fluctuation [8]. This necessity arises due to the cancellation of positive and negative components within the relatively high-frequency wave, encompassing 1/f modulation. In the context of solar flares, the demodulation process is envisioned to be facilitated by the threshold established through magnetic reconnection. The tiny energy required for magnetic reconnection may exhibit the 1/f fluctuation characteristic, aligning with the tiny energy associated with SFO that can trigger solar flares.

The energy associated with SFO may be significantly small compared with the total energy of a flare. Despite this, we believe that the SFO determines the 1/f fluctuation property of flares.

In general, a solar flare appears to involve two consecutive stages: (a) the gradual accrual of core magnetic energy manifested as accumulating intensity of magnetic fields with opposite polarities and (b) a subsequent tiny trigger that reconnects a very local strained magnetic fields initiating a sudden discharge of energy. From the preceding discussion, it is apparent that the 1/f fluctuation is closely associated with the second stage, (b).

The first stage, (a), is characterized by the progressive buildup of magnetic strain energy within many local domains of concentrated magnetic fields. This buildup phenomenon may be well described by applying the theory of self-organized criticality (SOC).

Regarding the second stage, (b), the final trigger, though minor, introduces sufficient energy to cause the reconnection of magnetic fields in a local area, overcoming the energy threshold and resulting in the burst release of magnetic energy. This minor final trigger, possibly linked to ongoing fluctuations of SFO on the solar surface, determines the timing of a solar flare; the characteristics of 1/f fluctuation in SFO may be inherited by the flares.

Moving forward, our analysis will focus more closely on this latter stage, (b), by applying the amplitude modulation theory to the analysis of 1/f fluctuations in solar flares.

## 4. Resonating Solar Five-Minute Oscillation

We delve into the potentiality of the solar five-minute oscillation (SFO) as a catalyst for 1/f fluctuation in solar flare activity. Specifically, our focus centers on elucidating how SFO eigenmodes contribute to the accumulation of frequencies, thereby generating low-frequency signals through amplitude modulation mechanisms.

The small displacement, denoted as u(t,r,θ,ϕ), of the solar atmosphere from its equilibrium position follows the Poisson equation

(1) $$\rho \ddot{u}=\kappa ▵u-\rho \nabla \phi_{g},\;\Delta \phi_{g}=4\pi G\rho$$

where $\rho ,\kappa ,G,\phi_{g}$ represent the mass density, bulk modulus, gravitational constant, and gravitational potential, respectively.

The stationary solution $u\left(t,r,\theta ,\phi \right)=v\left(r,\theta ,\phi \right)e^{-i\omega t}$ leads to the eigenvalue equation. Utilizing the variable separation method in the spherical coordinate system, we obtain a solution of the form

(2) $$u\left(t,r,\theta ,\phi \right)=R_{n,l,m}\left(r\right)Y_{l}^{m}\left(\theta ,\phi \right)e^{-i\omega_{n,l,m}t},$$

where $Y_{l}^{m}\left(\theta ,\phi \right)$ represents spherical harmonics. Modes are characterized by quantum numbers n=0,1,2,…, l=0,1,2,…, and −l≦m≦l. These modes are further categorized as pressure and gravitational modes. All parameters are uncertain depending on the detail of the solar interior, making the solution of the eigenvalue equation a complex task. Numerous numerical calculations and observational studies have been conducted on these eigenmode equations.

We utilize observational data pertaining to eigenmodes of solar oscillations obtained through helioseismology [15]. This dataset provides valuable information on numerous observed frequencies, disregarding the degeneration in the azimuthal order number parameter *m* (−l≤m≤l). A distinctive characteristic of these modes is the accumulation of frequencies towards smaller values of *l* for each *n* parameter, typically around $3\times 10^{-3}$ Hz. This property is pivotal for the emergence of 1/f fluctuation through the amplitude modulation mechanism [8].

To simulate the phenomenon, we randomly superimposed all sinusoidal waves with frequencies ranging from the lowest at 848.241 μHz up to 4669.16 μHz. The wave mode superposition is expressed as

(3) $$\Phi \left(t\right)=\sum_{k=1}^{N}\xi_{k}sin\left(2\pi \Omega_{k}t\right),$$

where $\xi_{k}$ is a random variable within the range [0,1], and N=2247 represents the total number of eigenfrequencies in the dataset. Subsequently, we conducted Fourier analysis (FFT) on the power spectral density (PSD) for the time series of the absolute value $\left|\Phi (t)\right|$. Notably, calculating the PSD for the bare Φ(t) yields no signal in the low-frequency domain. As the 1/f fluctuation signal is modulated in our model, a demodulation process is imperative; taking the absolute value is a typical demodulation method, essential for extracting 1/f fluctuations in the PSD analysis. The specifics of the demodulation process will be explored further in the subsequent discussion.

The PSD analysis results in a power-law with an index of approximately −0.42 within the low-frequency range of $2\times 10^{-5}$–$5\times 10^{-3}$ Hz, as illustrated in Figure 4 (left). However, it is noteworthy that the observed solar flare 1/f fluctuation occurs in the range of $2\times 10^{-8}∼10^{-3}$ Hz, considerably lower than our analysis. Further, the power index is definitely larger than in the case of the observed solar flare. These discrepancies suggest the need for a more nuanced consideration of realistic fine structures of the eigenstates and additional resonances, a facet we will delve into in the subsequent analysis.

Our analysis of resonance is currently incomplete, with several aspects requiring further exploration. Firstly, (a) each eigenmode is associated with a resonance curve, and numerous frequency-accumulating modes are linked to each mode. Secondly, (b) the degeneration in the azimuthal order number *m* should give rise to a fine structure around each principal frequency characterized by *n* and *l*. This degeneration in *m* is resolved by the solar non-spherical symmetry or the solar rotation. In this paper, we examine representative modes for both cases (a) and (b) to illustrate how the fine structure contributes to the emergence of 1/f fluctuation. A comprehensive analysis encompassing these aspects will be presented in our forthcoming publications.

To refine the power spectral density (PSD), we introduce the following effects: (a) each eigenfrequency labeled by *n* and *l* possesses a finite width, and (b) the degeneration in *m* is resolved by the solar rotation.

(a) Resonant modes are typically modeled by the Lorentzian distribution,

(4) $$R\left[\omega \right]=\frac{1}{{\left(\frac{\kappa }{2}\right)}^{2}+\left(\omega -\Omega \right){}^{2}},$$

where Ω is the fiducial resonance frequency, and κ characterizes the sharpness of the resonance. This function represents the frequency distribution density associated with the fiducial frequency Ω. The inverse function (tangent) of the cumulative distribution function (hyperbolic tangent) generates this distribution from the Poisson random field.

(b) Solar rotation resolves the degeneration in *m* by breaking the spherical symmetry of the system. Although the details are intricate, a rough estimate is provided by the resolved frequency [16,17] in the lowest perturbation in Ω/ω(≪1),

(5) $$\omega_{nlm}=\omega_{nl}+\frac{m}{l(l+1)}\Omega ,$$

where $\omega_{nl}$ is the degenerate eigenfrequency, and $\Omega =4.3\times 10^{-7}$ Hz is the frequency associated with the solar rotation. The coefficient of Ω is chosen approximately according to [16,17].

These effects are implemented through a specific process. Initially, we construct wave data by superposing *N* sinusoidal waves with eigenfrequencies after eliminating the degeneration in *m*. Additionally, we superimpose *M* resonant waves with frequencies proximate to the fiducial frequency, following the distribution in Equation (4). The fully superposed wave is defined as:

(6) $$\Phi \left(t\right)=\sum_{n=1}^{N}\sum_{i=1}^{M}\sin\left(2\pi \left(1+c\tan\left(\xi_{i}\right)\right)\Omega_{n}t\right),$$

where the parameter c=κ/Ω represents the relative line width for each eigenfrequency. The random variable $\xi_{i}$, ranging in [0,π/2], generates the frequency distribution through R(ω) in Equation (4). While *c* actually depends on each *n*, for simplicity, we use c=0.01. We utilized data [15], limiting *M* to 100 and *N* to 100.

**Figure 5:**
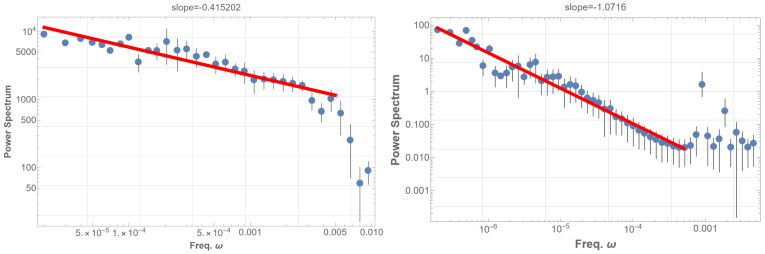
**left**: On the left side, the graph depicts the power spectral density (PSD) of the absolute value of the time sequence given by Equation (3), denoted as $\left|\Phi (t)\right|$. Here, Φ(t) represents the superposition of sinusoidal waves with the N=2247 eigenfrequencies of the solar five-minute oscillation (SFO), each with a random amplitude. Each mode is identified by the parameters n,l, and the azimuthal order number *m*, which is degenerate. Despite exhibiting a power law, this presentation barely demonstrates the characteristics of pink noise. **right**: On the right side, analogous to the left graph, this graph includes resonant modes and fine eigenmodes after resolving the degeneration in *m*. In constructing the data Φ(t), we superimpose sinusoidal waves with 100 frequencies from the lowest and introduce N=100 Lorentzian-distributed modes. The latter are randomly generated following Equation (4). The graph displays the PSD of the thresholded timing sequence of Equation (6), Φ(t). The threshold is set to select data points Φ(t) that surpass the mean although is moderately insensitive to the demodulation method. This presentation reveals nearly 1/f fluctuation with an index of −1.1 spanning over four digits. Notably, variations in thresholds and sample sizes in the PSD analysis consistently yield similar 1/f fluctuations. A red line in the low-frequency domain in the PSD graph represents the best fits of the data according to the least squares method within that domain.

As before, the power spectral density (PSD) of the bare Φ(t) exhibits no signal in the low-frequency region. However, taking the absolute value $\left|\Phi (t)\right|$ or applying arbitrarily set threshold data produces 1/f fluctuations (details in the caption of Figure 5). These operations essentially function as a demodulation of the original signal. Consequently, the 1/f fluctuation becomes evident only after demodulation and proves to be quite robust. Figure 5 (right) illustrates the PSD of the thresholded data, demonstrating an approximate 1/f fluctuation with a power index of −1.1, covering a frequency range extended down to $2\times 10^{-7}$ Hz. This range partially coincides with the observed range below $10^{-3}$ Hz. In our future study, further refinement of the PSD analysis is intended, incorporating finer structures of eigenfrequencies, decay times, and deviations of the Sun from spherical symmetry. The introduction of gravitational modes, alongside pressure modes, which operate in much lower frequency domains, is also of interest.

In the preceding discussion, we superimposed the eigenmodes of the solar five-minute oscillation (SFO) to obtain amplitude modulation and pink noise, explaining the observed 1/f fluctuation in solar flares. However, a more direct examination of the bare data of SFO before decomposition into eigenmodes is warranted. This implies that the resonance of SFO directly yields pink noise. Initially, we utilize SOHO-GOLF data on the fluctuations of the time elapsed T(t), for the acoustic waves to travel through the solar center [18] measured in the unit second. Details are expounded in [19], with the data spanning about 16.5 years and an interval of 80 s, albeit with some data-missing periods.

**Figure 6:**
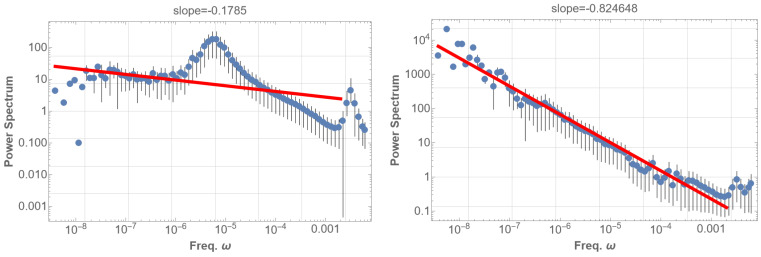
(**left**) The power spectral density (PSD) is presented for the SOHO-GOLF 16.5-year data, focusing on the time elapsed T(t) for the waves circumnavigating the Sun [18]. The rightmost peak corresponds to the typical five-minute mode of the solar five-minute oscillation (SFO) at $3\times 10^{-3}$ Hz. (**right**) The PSD is shown for the absolute value of the original data, revealing 1/f fluctuation with a slope of −0.82 over approximately six digits. In both figures, the frequency region corresponding to SFO is deliberately excluded from the fitting region of 1/f fluctuations. This exclusion is based on our consideration that the 1/f fluctuation originates from the wave beat produced by the resonating SFO; such a beat typically manifests in a lower frequency domain than the resonator itself. Practically, including the SFO frequency region in the analysis is not expected to influence the results significantly. A red line in the low-frequency domain in the PSD graph represents the best fits of the data according to the least squares method within that domain.

We commenced by calculating the power spectral density (PSD) of the original data T(t). The result is depicted in the left graph of Figure 6. A prominent peak appears around $3\times 10^{-3}$ Hz, corresponding to the typical five-minute mode of solar oscillation. From there, a partial power-law behavior is observed toward $6\times 10^{-6}$ Hz with an index of about −1, followed by a flat behavior at lower frequencies. This partial 1/f fluctuation may stem from instrumental origins [20], potentially not reflecting genuine solar properties.

Conversely, when taking the absolute value of the data, the 1/f fluctuation region in the PSD extends toward the lowest frequency limit, as illustrated in Figure 6 (right). The behavior of the SOHO-GOLF data T(t) is precisely the same as the sound data of orchestra music [21] or the sound of a big bell. While the sound wave amplitude time-sequence data do not exhibit pink noise, the square of the amplitude showcases apparent pink noise. These operations of squaring or taking the absolute value naturally correspond to the demodulation process, revealing the encoded pink noise.

This forms part of the rationale behind our belief that solar eigenoscillations contribute to the 1/f fluctuation observed in solar flares. To extract 1/f fluctuation from the wave T(t), we required operational demodulation, such as taking the absolute value, due to the low-frequency 1/f fluctuation being encoded as amplitude modulation. In contrast, operational demodulation was not necessary in the case of solar flares, as the demodulation process is considered intrinsic to the Sun and is already facilitated by magnetic reconnection.

An alternative analysis involves ground-based data from BiSON [22,23], measuring radial velocity from January 1985 to January 2023 (all sites, optimized for quality). The PSD of the original bare data does not exhibit pink noise. However, taking the absolute value of the original data results in clear 1/f fluctuation with a slope of −0.71, although this index is slightly larger than the right side of Figure 6. This discrepancy is likely influenced by artificial peaks corresponding to the periods of the Earth’s spin and rotation at $1.1\times 10^{-5}$ Hz and $3\times 10^{-8}$ Hz.

A variety of demodulation methods have been considered in association with this BiSON data manipulation. In our previous analysis, we focused on the absolute value of the data, but it is noteworthy that other manipulations can also be employed to extract pink noise. For instance, the squared data exhibit pink noise, while the 1/f fluctuation disappears in the cubed data. However, when the data is raised to the fourth power, 1/f fluctuation reappears. These findings strongly indicate that the amplitude-modulated 1/f fluctuation emerges after specific demodulation processes.

Similar phenomena are often observed in sound systems. For example, we examined sound data collected at the water-harp cave (Suikinkutsu) at HosenIn Temple in Kyoto [24]. The sound is generated by the perpetual impact of water drops on the water surface in the two-meter Mino-yaki pot underground [25]. Although the original sound data barely show pink noise, the squared data from this sound source clearly display 1/f fluctuation with an index of −0.80 for four digits. The resonator, in this case, is presumed to be the Mino-yaki pot.

## 5. Timing Statistics

In our analysis of Section 2, we identified 1/f fluctuation in the time series of solar flare timing and speculated that this characteristic might be indicative of the low-energy trigger for solar flares. Similar pink noise behavior is observed in seismic activity [9], where time series of earthquake occurrences is often described by the Weibull distribution function [26,27]:

(7) $$f\left(x\right)=\frac{\alpha }{\beta }{\left(\frac{x}{\beta }\right)}^{\alpha -1}\exp\left(-{\left(\frac{x}{\beta }\right)}^{\alpha }\right).$$

This leads us to explore whether the solar flare timing sequence is characterized by the Weibull distribution and its potential connection to pink noise.

**Figure 7:**
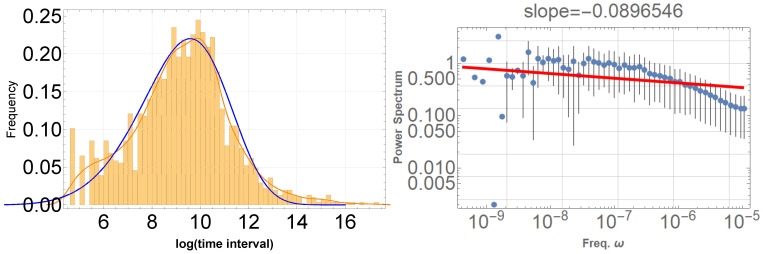
(**left**) Orange bars indicate the frequency distribution of the logarithm of the time intervals between solar flare occurrences, while the thin red line represents the smoothed version of the fitting curve. The blue line denotes the Weibull distribution with the parameters α=5.3 and β=9.9, best fitting the solar flare data. (**right**) The PSD is presented for artificial data with random time intervals generated by the best-fit Weibull distribution. It is evident that the PSD of these artificial data is flat, indicative of a random distribution. A red line in the low-frequency domain in the PSD graph represents the best fits of the data according to the least squares method within that domain.

Upon examination of the GOES data [12] used in our analysis, we discover that, the logarithm of the time intervals between solar flares follows the Weibull distribution, as illustrated in Figure 7 (left). The best-fit parameters are α=5.3 and β=9.9. Therefore, similar to seismic activity, the statistical distribution of solar flare timing can be effectively characterized by the Weibull distribution. The question then arises: to what extent does this Weibull distribution characterize the 1/f fluctuation property?

We have checked that a time sequence simply following the Weibull distribution does not exhibit pink noise; the power spectral density (PSD) becomes flat in the low-frequency range, as depicted in Figure 7 (right). Consequently, the 1/f fluctuation observed in solar flare timing is independent of the Weibull distribution, as the behavior observed in seismic cases. This property is readily understood; the time sequence, constructed with randomly chosen intervals according to Weibull distribution statistics, lacks long correlation times. In contrast, 1/f fluctuation inherently possesses long correlation times, thereby manifesting as a low-frequency property.

## 6. Robustness and Inherited 1/f Fluctuation

We have delved into the origin of 1/f fluctuation in solar flares, applying the overarching concept that the accumulation of frequencies in numerous waves leads to beat or amplitude modulation. Frequency accumulation is achieved through resonance, and we have successfully explored this concept using the solar five-minute oscillation (SFO), which are consistently resonating. It is plausible that magnetic reconnection serves as the demodulation (DM) of this amplitude modulation (AM), resulting in 1/f fluctuation in solar flares. If this holds true, the combination of SFO (as AM) and magnetic reconnection (as DM) may generate 1/f fluctuation beyond solar flare in other extended regions of the solar neighborhood.

For instance, if magnetic reconnection induces solar wind through jetlets [28], triggered by SFO, then the solar wind [29] may also exhibit pink noise. Indeed, 1/f fluctuation in the solar wind has been observed over many years [30,31,32]. SFO has been detected in the solar corona [33], suggesting the potential observation of 1/f fluctuation in that context as well.

Furthermore, the solar wind may interact with the Earth’s atmosphere, inducing chemical reactions leading to the production of the ${\mathrm{NO}}_{3}^{-}$ isotope. This isotope could then become embedded in Antarctic ice cubes. Research in this area is ongoing. If solar wind and solar flares influence the Earth’s surface, then sea surface temperature may also exhibit 1/f fluctuation, similar to the case of seismic activity.

In seismic activity, which also displays pink noise, seismic events inherit a 1/f fluctuation pattern, potentially resulting from amplitude modulation due to resonance with Earth free oscillation (EFO) in the lithosphere [9]. This EFO may further contribute to the 1/f fluctuation observed in the time sequences of volcano eruptions and the fluctuation of the Earth’s rotation axes.

Including the above robustness of pink noise, we compare solar flares and earthquakes in Table 1. This table is preliminary and will be finalized in our forthcoming study.

## 7. Conclusions and Prospects

In conclusion, our investigation has identified 1/f fluctuation in the solar flare time series, and we have partially elucidated its origin by applying our proposed mechanism: 1/f fluctuation emerges from the resonance of the solar five-minute oscillations via amplitude modulation and demodulation.

Our GOES data analysis of the solar flare time sequence has revealed distinct low-frequency properties. Specifically, the power spectral density of low-energy flares ($E\leq E_{mean}$) exhibited 1/f fluctuations, while high-energy flares ($E>E_{mean}$) displayed a flat spectrum. Notably, the time sequence of flare occurrences demonstrated clearer 1/f fluctuations, indicating that low-energy characteristics play a pivotal role in triggering the observed 1/f fluctuations in solar flares. Building on our recent proposal that 1/f noise arises from amplitude modulation and demodulation, we postulated that this modulation is encoded through resonance with the solar five-minute oscillation (SFO) and demodulated via magnetic reconnection.

To test this hypothesis, we constructed a dataset by superposing sinusoidal waves with 2247 eigenfrequencies of SFO. The absolute value of this time sequence marginally exhibited 1/f fluctuations with a power index of −0.42 down to $2\times 10^{-5}$ Hz. Further refinement of the data, considering resonance effects and finer structures labeled by *m* induced by solar rotation, involved adding 100 extra modes generated by resonant Lorentzian distributions for the first 100 eigenfrequencies of SFO after resolving the degeneration in m. The absolute value of this refined time sequence clearly displayed 1/f fluctuations with a power index of −1.1 down to $10^{-7}$ Hz, largely overlapping with the observed range of solar flare 1/f fluctuations (power index −1.0 from about $2\times 10^{-3}$ Hz down to $2\times 10^{-8}$ Hz). Thus, our analysis provided partial verification that SFO triggers seismic 1/f fluctuations.

Further investigation into SOHO-GOLF data and BiSON data for velocity fluctuations in the solar atmosphere revealed that the original time sequence of these data barely exhibited 1/f fluctuations, while the absolute values of the time sequence did display clear 1/f fluctuations. This lends additional support to our proposition: SFO is the origin of solar flare 1/f fluctuations.

Additionally, our examination of the time sequence of solar flare occurrences revealed adherence to a Weibull distribution. However, an artificial time sequence composed from the Weibull distribution barely exhibited 1/f fluctuations, suggesting that the Weibull distribution does not fully characterize solar 1/f fluctuations.

Lastly, our comparison of 1/f fluctuations in solar flares and earthquakes, as demonstrated above, has revealed remarkable similarities between them [34,35]. The underlying commonality in these phenomena is the generation of 1/f fluctuations through amplitude modulation by spherical resonators in the universe, such as stars and planets. This concept can naturally extend to other celestial objects, including black holes and neutron stars. Specifically, we have conducted a preliminary analysis of six years of MAXI X-ray data from Cyg X-1, obtaining 1/f fluctuations with a power index of −0.8 over four orders of magnitude. We hypothesize that the resonator in this case is the quasi-periodic oscillation of a black hole. Additionally, the similarity of repeated Fast Radio Burst (FRB) data with solar flares and earthquakes is noteworthy. It would be intriguing to explore whether this signal is related to the eigenoscillations of neutron stars or magnetars. These analyses will be detailed in our forthcoming separate papers. This broader perspective underscores the potential universality of the proposed amplitude modulation and demodulation mechanism across diverse astrophysical phenomena. 

## Figures and Tables

**Table 1:** Similarity of solar flares and earthquakes from the view point of pink noise. This is a tentative table, and the detail will be reported soon by the authors.

	Solar Flare	Earthquakes
AM (resonator)	Solar five-minute oscillation (SFO)	Earth free oscillation (EFO)
DM	magnetic reconnection	fault rupture
PSD total data	flat (GOES16, RHESSI)	flat (USGS)
PSD low-energy	pink (GOES16, RHESSI)	pink (USGS)
PSD timing	pink (GOES16, RHESSI)	pink (USGS)
PSD superposed eigenmodes	pink (JSOC)	pink (T. G. Masters, R. Widmer)
Weibull distribution	yes: α=5.3 and β=9.9 (GOES16)	yes: α=6.3 and β=7.63 (USGS)
PSD resonator	pink (SOHO-GOLF, BiSON)	?
inherent phenomena	solar wind, sunspot number, nitrate, SST, cosmic ray	volcano eruption, rotation axes

## Data Availability

The data presented in this study are available on request from the corresponding author.
